# Food Safety Behaviours among Food Handlers in Different Food Service Establishments in Montenegro

**DOI:** 10.3390/ijerph20020997

**Published:** 2023-01-05

**Authors:** Snežana Barjaktarović Labović, Ivana Joksimović, Igor Galić, Miro Knežević, Marijana Mimović

**Affiliations:** 1 Institute of Public Health, John Jackson bb, 81 000 Podgorica, Montenegro; 2 Clinical Centar Podgorica, Ljubljanska bb, 81 000 Podgorica, Montenegro

**Keywords:** food handlers, hygiene, food safety, training, food establishments

## Abstract

Foodborne diseases in food facilities are a major public health problem, due mostly to the limited surveillance and educational level of food-handling workers. This study was conducted in 220 food service locations in Montenegro. Participants’ behaviour was assessed by a survey using the specifically designed structured questionnaire, administered before and after the training. To determine the effect of the training on the performance of food handlers, a microbiological analysis of food contact surfaces and food handlers’ hands was also performed. The behaviour of food handlers, viewed as a whole, is unacceptable. There was a statistically significant difference (<0.05) among participants who completed catering school compared with those who did not, regarding hand washing. The type of facility in which participants worked (restaurant, bakery, or pastry shop) revealed statistically significant differences (<0.05) in relation to hand washing, that is, restaurant employees had better habits than those from bakeries and pastry shops. Before the training, participants showed acceptable behaviour regarding hand hygiene, but it was much better after the training. Results of microbiological analyses of food contact surfaces and food handlers’ hands indicated better results after the education, especially with regard to hand swabs. The results of this study indicate the importance of education to improve food handling practices among food handlers, which might also decrease the possibilities for contamination of food.

## 1. Introduction

Foodborne illnesses remain a top public health concern despite numerous preventive measures and controls implemented in the food production value chain [1,2,3]. In 2020, the majority of reported cases in the EU counties (60.9%) were linked to restaurants or institutional food-service facilities [4]. In Montenegro [5], of 30 foodborne outbreaks reported between 2016 and 2020, more than 25% were linked to catering establishments alone.

Montenegro is a popular summer tourist destination, where the increasing number of tourists each year surpasses the nations’ population [6], and more than a half of all catering services are in operation only from May to September.

The food-service facilities that operate only during the tourist season employ workers who often have no experience working with food and who come from different cultural backgrounds. Seasonal workers are often not professionally trained and, due to lack of knowledge, are not aware of the importance of food safety nor do they know the consequences of improper food handling in these facilities. An additional problem is that owners of seasonal facilities rarely invest in staff training, as employees change from year to year.

It is widely recognized that inadequate food preparation practices and food service of community kitchens and dining rooms are strongly associated with poor microbiological quality of served foods [7,8,9,10].

Numerous foodborne outbreaks occur due to improper food handling practices [11,12]. The knowledge and practices of food handlers are essential to protect consumer health, especially vulnerable populations that are more susceptible to infections [13,14]. Food handlers may contaminate food through improper hand hygiene, inadequate sanitation of equipment and surfaces, or inadequate food preparation and holding [11]. Improper handling of food and non-compliance with hygiene procedures are considered the main cause of foodborne diseases linked to food service [14].

Food safety training is a recognized strategy for preventing foodborne outbreaks in the food-service industry. For example, a comparison between restaurants in which outbreaks had occurred and those without outbreaks, it has been shown that qualifications of kitchen managers, including food safety training, were critical in preventing the outbreaks [15,16]. The results of a study conducted in Kenya point to food safety training as an appropriate means of improving the knowledge and hygienic practices of food handlers [17]. Education of food handlers is a preventive measure that leads to the reduction of food safety risks.

The main objective of this study was to investigate the effect of the educational interventions on the handling practices of food handlers working in different food services establishments in Montenegro. The secondary objective of the study is to examine the hygienic conditions in the kitchens of catering establishments before and after training by analysing samples of food contact surfaces and food handlers’ hands.

## 2. Materials and Methods

### 2.1. Study Area

A study was conducted from February 2016 to February 2018, in 220 randomly select-ed food services sites from six cities at the Montenegrin coast, namely Ulcinj, Bar, Budva, Tivat, Kotor, and Herceg Novi. A two-stage proportionally stratified random sample was used to select participants.

The frame for the sample was the list of permanent catering establishments registered in the Department for Food Safety of the Ministry of Health of Montenegro in 2015. In six cities of the Montenegrin coast, 697 permanent catering establishments have been registered, namely 322 restaurants (pizzerias, taverns, and fast-food establishments), 127 bakeries, 42 pastry shops, and 206 other facilities that have not been included in the study, as they do not prepare or serve food (cafes and cafe/bars). After listing all catering facilities in the coastal cities, facilities to be included in the study were randomly selected, and a total of 60 restaurants, 30 bakeries, and 20 pastry shops were selected for the experimental group and included in the study.

According to the data of the Food Safety Department of the Ministry of Health of Montenegro, in 2015, 3532 people worked in permanent catering establishments. The survey included 385 food handlers, which represents 10.9% of this population, with 210 participants from restaurants, 105 from bakeries, and 70 food handlers from pastry shops. All participants had direct contact with food and surfaces in contact with food, including activities such as receiving, storing, preparing, or serving foods.

### 2.2. Research Instrument—Questionnaire

For the purpose of this study, a specifically designed structured questionnaire was designed on the basis of the previously published articles [18,19,20,21,22,23].

The first part of the questionnaire contained questions related to the demographic characteristics of the participants, such as gender, age, level of education, work experience, and current position in the hospitality industry. The part of the questionnaire related to behaviours, personal hygiene, and hand hygiene, included a total of 12 statements on a five-point Likert scale, ranging from “Never” to “Always” (1→5). For each question, the percentage of correct practices was calculated according to the number of respondents answering that they “always” perform specific hygiene activities. However, when they were asked questions related to using a towel to wash their hands or questions regarding eating, drinking, or smoking during food preparation and handling, the percentage of correct answers was related to the number of respondents answering “never”. On the basis of the obtained calculated scores, the behaviour of the participants was classified into 3 categories, namely acceptable behaviour (85–100%), marginally acceptable behaviour (75–84%), and unacceptable behaviour (<74%). The validity of the questionnaire was checked by a pilot study conducted with 30 participants employed in catering facilities in Bar.

As a result of the pilot study, several questions from the questionnaire were dropped and some were modified to make them clearer. Participants of the pilot study were not included in the research. Using Cronbach’s Alpha coefficient, the reliability of the test was high and amounted to 0.90.

Informed consent was obtained from each to participate in the intervention. Ethical approval for the study was obtained from the Institute of Public Health Podgorica No. 01-2882/2 from 29 April 2015. Each participant completed the survey three times. The initial assessment was conducted before the food safety training (Section 2.4), and then again four weeks and six months after the training.

The survey was performed three times, with initial assessment which was performed before the training (Section 2.4), then again four weeks and six months after the training. For that purpose, the same questionnaire was used to determine the impact of training on the changes in food handlers’ behaviour. At the same time, samples for microbiological analysis (Section 2.3) were taken before the training, then four weeks and six months after the training.

### 2.3. Microbiological Analysis

To determine the effect of the training on the microbiological quality of food contact surfaces and equipment and hand hygiene, samples were taken for the microbiological analysis before the training and four weeks and six months after the training. At each sampling day, a total of 90 swabs were taken from each food establishment, including 30 samples of food contact surfaces, 30 samples of equipment, and 30 samples of food handlers’ hands. These samples were taken from the control group of food establishments in which training was not given, as well as from the examined group to whom training was given.

The food contact surfaces included a clean board for preparing meat, and the samples of cleaned equipment and utensils were collected to determine the effect of cleaning and disinfection. In addition, food handlers’ hands were swabbed, and these handlers from the examined group also filled out the questionnaire (Section 2.2). All samples for microbiological analysis were taken in accordance with the international standard ISO 18593. During sampling, the area to be sampled was determined and then swabbed. The swab was dipped into a tube containing sterile dilution fluid (buffered peptone water) and mixed. The samples were transported in a refrigerated transport box set from 1 °C to 4 °C, which arrived at the laboratory within 4 h. Microbiological analyses were performed in the laboratory of sanitary microbiology of the Institute of Public Health in Podgorica, accredited in accordance with ISO 17025. Samples of food contact surfaces, utensils, and food handlers’ hands were analysed for the total bacterial count (TBC), following international standards ISO 4833:2007 by growing bacteria onto agar plates, “Plate Count Agar” (PCA), and ISO 21528-2 method for the detection and enumeration of *Enterobacteriaceae* by growing bacteria onto Violet Red Bile Glucose (VRBG) agar, respectively. All procedures are carried out in accordance with the Regulation on microbiological criteria for food (Official Gazette of the Republic of Montenegro, No. 26/16), which coincides with Commission Regulation (EC) No 2073/2005, as well as the criteria used to determine acceptable results of microbiological analysis given in the Guidance for microbiological criteria issued in 2012 (Official Gazette of Montenegro, No. 53/12). More specifically, for metal, glass, and porcelain surfaces, the acceptable samples were those with TBC ≤ 10 CFU/cm^2^; for utensils, acceptable was considered as TBC ≤ 100 CFU/cm^2^; and for food handlers’ hands, acceptable samples were those with TBC ≤ 200 CFU/cm^2^. Regarding the number of *Enterobactericeae*, for all three types of samples, values of 0–1 CFU/cm^2^ were classified as acceptable values, while unacceptable results were obtained when the number of *Enterobactericeae* is >1 CFU/cm^2^.

### 2.4. Food Handlers’ Training

In the second phase of the research, food handlers’ trainings were conducted by a hygiene specialist in the examined group. Trainings were performed in small groups with a maximum of 12 participants. Different teaching techniques were employed, including video recordings, audio recordings, short films, presentations, flyers, and quizzes. Each module was created with a duration of 180–240 min. The modules covered the following issues: (i) personal hygiene, foodborne illnesses, and good hygiene practices; (ii) incoming control, traceability, and food analysis; and (iii) temperature control, control measures, and documentation.

### 2.5. Data Analysis

Data were analysed using SPSS version 20 (IBM, Armonk, NY, USA). For the description of socio-demographic characteristics, descriptive statistics were used; mean values and standard deviation (SD) for numerical and normally distributed data were calculated, while for categorical data, percentages (%) were calculated. Cross-tabulation was carried out to examine the distribution and relationship of the variables. We used regression analysis to determine a connection between the knowledge and behaviour of the respondents. We compared average value levels with correction for SD. Regression analysis was performed for differences in the results of swabs four weeks and six months after the training. Non-parametric χ^2^ test was used for the determination of statistically significant differences among the different variables. The value of *p* < 0.05 was considered statistically significant.

## 3. Results

### 3.1. Sample Characteristics

This study involved a total of 385 food handlers. A total of 52.2% participants graduated from elementary and high school, whereas 14.8% participants had either a college or university degree. The greatest number of respondents (70.1%) had a secondary school (standard school curriculum) education, 57 participants (14.8%) had a higher level of education, and 58 (15.1%) had a primary level of education (8 years of elementary school). Participant characteristics are shown in Table 1.

### 3.2. Food Handling Practices before the Training

Observed as a whole and scored in relation to the defined criteria, the behaviour of the food handlers has been mostly unacceptable, with the mean values of 71.5 ± 22.0. A detailed overview of obtained results is shown in Table 2. Obtained results indicated unacceptable behaviour mainly in relation to hand hygiene. A little more than half (55%) of participants indicated that they wash their hands properly, and 44.2% do not use a towel to wipe their hands after washing, while 45.2% regularly disinfect their hands after washing. Slightly less than three-quarters of the participants (71.7%) regularly wash their hands before contact with raw foods; only 67.3% use special utensils for heat-treated food; after touching the hair, 66.5% wash their hands, while 71.4% of participants take off their jewelry before starting to work with food. While working with food, 72.5% of them do not eat or chew gum, and 72.7% regularly wear headgear. Marginally acceptable behaviour is recorded for participants who wash their hands after contact with raw foods (77.4%). Acceptable behaviour was recorded in 88.1% of participants who cover their nose or mouth when sneezing and coughing, as well as 88.8% who do not consume cigarettes during work.

Gender, age, years of service, education, and position in which they work (workplace) did not prove to be determinants in relation to the behaviour of participants when handling food (*p* > 0.05). Completed or unfinished catering school and type of facility (restaurant, bakery, or pastry shop) are not statistically significant in relation to the behaviour (Table 3, *p* > 0.05). There is a statistically significant difference among participants who completed catering school compared with others regarding hand washing after touching the face or hair (*p* < 0.05). The behaviour of food handlers depended on the type of facility in which they work (restaurant, bakery, or pastry shop). When washing hands, food handlers in restaurants have slightly better habits compared with those from bakeries and pastry shops (*p* < 0.05). In addition, food handlers in restaurants were more up-to-date in hand washing before having contact with raw foods (*p* < 0.05). In addition, they were more diligent in using special utensils for cooked and fresh food (*p* < 0.05).

### 3.3. Food Handling Practices after the Training

The results of food handlers’ behaviour four weeks after the training showed that the training was very useful for improving the behaviour of food handlers. However, the results obtained six months following the training showed that food handlers relaxed over time. Still, the results obtained six months following the training were significantly improved compared with their behaviour before the training (*p* < 0.05).

The greatest improvement was observed in maintaining hand hygiene. Before the training was conducted, 55.1 ± 0.9% of food handlers properly washed their hands; this value was 98.2 ± 0.2% four weeks after the training, and 97.6 ± 0.5% of food handlers properly washed their hands six months after the training (Table 4).

### 3.4. Microbiological Results of Work Surfaces, Equipment, and Food Handlers’ Hands

#### 3.4.1. Results Obtained in the Restaurants

In the first sampling, the largest number of improper swabs belonged to the employees’ hands (in the examined group, it was 50.0% and in the control group, 46.6%, Table 5). After the training, slightly better results were recorded in the examined group (second sampling, 66.6% and third sampling, 56.6%), while in the control group, approximately the same percentage of acceptable results was recorded. For the work surfaces, similar results were obtained. In the first sampling, 66.3% in the examined group and 63.3% in the control group were acceptable. In the second and third samplings, this was 80.0% and 73.3%, respectively, in the examined group, while in the control group, 63.3% and 66.6% were acceptable in the second and third samplings, respectively. The situation was similar with equipment swabs. In the first sampling, 63.33% of the samples in the examined group and 63.33% in the control group were acceptable. After the training was performed, in the second sampling period, 73.3% was acceptable for the examined group and 63.3% for the control group, while in the third sampling, 70.0% and 56.6% were acceptable for the examined and control groups, respectively (Table 5).

#### 3.4.2. Results Obtained in the Bakeries

The microbiological results of the samples taken from the bakeries were different from those obtained in the restaurants (Table 6). In the first sampling period, only 36.6% from the examined group and 30.0% in the control group were acceptable.

In the second sampling period, we determined 66.7% of the acceptable results for food handlers’ hands in the examined group, while only 33.3% in the control group. In the third sampling period, 63.3% of the samples in the examined group and 36.6% in the control group were acceptable. Regarding the results for work surfaces, 56.6% of samples in the examined group and 60.0% in the control group were acceptable. In the second sampling period, 76.6% of the samples were acceptable, and in the control group, this was 60.0%.

After the third sampling period, 73.3% of the swabs in the test group and 43.3% in the control group were acceptable. In the case of equipment after the first sampling, 43.3% of swabs were acceptable in the examined group and 36.6% in the control group. After the training was given to the food handlers, 70.0% of swabs were acceptable in the examined group and 33.3% of swabs in the control group. In the third sampling period, 60.0% of swabs were acceptable in the examined group, while this was 43.3% in the control group.

#### 3.4.3. Results Obtained in the Pastry Shops

The obtained results indicated the smallest percentage of acceptable results in the samples taken from the pastry shops (Table 7). During the first sampling period, only 36.6% of samples of work surfaces were acceptable in the examined group, while this was 36.6% in the control group.

During the second sampling period, in the examined group, 76.6% of samples were acceptable, while in the control group, this was 43.3%.

In the third sampling period, 73.3% were acceptable in the examined group, and 56.6% in the control group (Table 7).

Regarding the equipment used in the pastry shops, 60.0% of swabs were acceptable in the examined group, and 46.6% in the control group. During the second sampling period, 73.3% of swabs were acceptable in the examined group, and 53.3% in the control group. In the third sampling period, 66.6% of swabs were acceptable in the examined group, and 46.6% in the control group.

Regarding the results obtained for the food handlers’ hands in the examined group, 40.0% of swabs were acceptable, while in the control group this was 33.3%. In the second sampling period, in the examined group, 66.65% samples were acceptable, while in the control group, it was only 40.0%. In the third sampling period, 63.3% of swabs were acceptable in the examined group, while 30.0% were acceptable in the control group.

Regression analysis determined differences in the results of swabs taken four weeks (second period of sampling) and six months (third period of sampling) after the training. Employee education and training resulted in better food handlers’ behaviour, which has indirectly improved the hygiene in the food environment, including better hygiene of work surfaces, equipment, and hand hygiene (Table 8).

The relationship between the microbiological results of swabs before and four weeks after training was determined, as well as the relationship between the results obtained four weeks and six months after the training. These results are shown in Table 9.

## 4. Discussion

Similar to previous studies, the results of this study indicated the importance of the human factor as a potential cause of contamination, especially in relation to food contamination by dirty hands. The results of our research showed that approximately half of the participants (55%) wash their hands properly, and 44.2% do not use towels after washing hands, while 45.2% regularly disinfect their hands after washing. Slightly less than three-quarters of participants (71.7%) wash their hands regularly before contact with raw foods, while after contact with raw foods, this is 77.4%. In Greece, 95.6% wash their hands after processing raw meat, 99.1% in Serbia, and 100% of participants in Portugal do so [24]. More than 67% of participants used special utensils for heat-treated food, but one-third do not do so and do not act responsibly.

A study conducted by Akabanda et al. [25] in Ghana reported that 73% of employees who work with food wash their hands before cooking or serving food. When washing hands, 92.4% use antibacterial soap, and 7.2% prepare food even when they feel sick. In contrast to this research, the results obtained in Slovenia showed that 97% of participants wash their hands properly [26]. It was determined that 66.5% of participants wash their hands after touching their hair, 71.4% of the participants take off their jewelry before working with food, and 72.5% of them do not eat or chew while working with food, while 72.7% regularly wear headgear. Result of study by Smigic at al. [27] show that 95% of students apply good practices of hand hygiene before preparing food. Hygienically acceptable behaviour was found in 88.1% of our participants, who covered their nose and mouth when coughing and sneezing. One-tenth of the participants, despite the ban on tobacco consumption in workplaces, still partake at the workplace and in rooms where food is prepared. Completed or unfinished catering school and the type of facility in which they work (restaurant, bakery, or pastry shop) did not prove to be statistically significant in relation to the knowledge that participants have regarding hygiene and food safety. Participants with a degree in catering have better hygiene habits. The behaviour of employees differs in certain segments depending on the type of facility in which they work (restaurant, bakery, or pastry shop). When washing hands, employees in restaurants have slightly better habits compared with those from bakeries and pastry shops (*p* < 0.05). In addition, employees in restaurants were more up-to-date in washing their hands before having contact with raw foods (*p* < 0.05). In addition, they were more diligent in using special utensils for heat-treated and fresh food (*p* < 0.05).

A study conducted in China analyzed the knowledge of employees in university restaurants [28]. The study included those who did not have any type of training in the years before the research. Participants showed a high level of knowledge about the importance of hand hygiene, as well as knowledge that fresh and heat-treated food should be treated separately and with separate utensils. It is interesting to find that better food safety knowledge of the participants did not contribute to better food safety behaviour [29]. Osalili et al. [30] examined food safety knowledge of food-service staff in food establishments at the universities in Jordan. A total of 520 food-service participants from 79 food-service establishments located at 27 universities (34 campuses) were conveniently selected to participate in this cross-sectional study. The highest score of correct answers refers to questions related to respecting the principle of maintaining personal hygiene (74.9%). Before preparing meat, 96.5% wash their hands, after contact with raw meat (93.3%), while 90.8% wash their hands if they touch their face or hair while working with food. If workers touch their clothing during food processing, 74.8% wash their hands, while 31.9% wash their hands properly. Approximately 90% identified eight situations in which they wash their hands regularly. Approximately 73% of participants wash their hands after touching money, 74.8% wash their hands if they touch their work clothes during food preparation. Only 32% of participants knew how long it would take to wash their hands properly. Participants in an investigation of the level of food safety knowledge in food establishments in three European countries by Smigic at al. [24] were aware of the requirement for washing hands after handling waste (98.4%), using bathroom facilities, handling raw food, or blowing their noses (97.0%), or eating and drinking (90.4%). Studies performed by Aarnisalo at al. [31] and Todd et al. [12], as well as a number of other papers, also emphasize the importance of maintaining personal hygiene and especially emphasize the importance of hand hygiene in pathogen transmission. Some researchers, including Todd and co-workers, pointed out that proper hand hygiene is more important than cleaning and disinfecting work surfaces in food preparation facilities in order to prevent the spread of pathogenic microorganisms.

In Sudan, research was conducted in 21 restaurants in seven different areas [32]. All participants agree that washing hands before starting food preparation, wearing gloves during food preparation, as well as proper cleaning and handling of food preparation equipment reduces the risk of food contamination. People who have wounds on their hands and changes on the skin should not touch food without gloves. They also agree that it is necessary to wear headgear while handling food. This study found a positive correlation with food hygiene practice and knowledge of food safety. In the study by Barjaktarovic et al. [33], the average level of knowledge of maintaining hygiene in food facilities was 68.6%. Participants had a high level of knowledge (93%) that objects and surfaces in contact with food should be first cleaned and washed and then disinfected. However, almost one-third of the participants (28.9%) did not know that cleaning and disinfection are not the same procedure. Two weeks after training, the number of correct answers was significantly higher; thus, knowledge of employees increased from 59.1 ± 24.7 to 91.9 ± 5.3.

The importance of maintaining personal hygiene is recognized by 92.5% of hotel employees in Poland, although their behaviour does not correlate with knowledge. More than half of participants (60%) know how often protective clothing should be changed, while 98% know that if a person who handles food has *Salmonella*, such a person should not come into contact with food. Almost 90% of them knew that hands should be washed after handling food, while 35% state damage to nail polish as a risk of contamination, and 63.5% believe that wearing jewelry is a risk for food contamination. Regarding washing, 84.5% of participants do not wash their hands properly, while 8% think that it is enough to wash their hands with soap and dry them with a paper towel, and 5.5% think that they should wipe their hands with a towel after washing. More than half of the participants (56.5%) do not know how to act properly if a person is cut with a knife. They believe that it is enough to protect a wound or injury on the skin with a bandage. In addition, slightly more than one-third of the participants (35.0%) in this research believe that education about safe food is important. Half of the participants stated that they had undergone some type of training [34]. A survey of knowledge, attitudes, and behaviour of employees in 29 restaurants in Ghana [25] showed that the majority of food-handlers in this study knew the importance of general sanitary practices, such as regular hand-washing at the work place (98.7% correct answers), wearing of gloves (77.9% correct answers), proper cleaning (86.4% correct answers), and detergent use (72.8%). Participants also agreed that individuals with abrasions or cuts on their fingers or hands should not touch unwrapped foods (87.2%). The majority (88.1%) of food-handlers were aware that food should not be handled with long and painted fingernails. In assessing the food safety practices of the institutional food-handlers, 88.1% reported that they do not use gloves during the distribution of unpackaged foods. A majority (61.7%) of the food-handlers do not use aprons or wear masks when necessary. Additionally, they eat and drink during working hours. Among them, 61.7% reported that they do not use sanitizer in washing utensils such as plates, mugs, and spoons. A study conducted in Ghana found that 77.9% of participants wore headgear during food preparation.

Our participants have a higher level of knowledge in relation to the fact that people with wounds on their hands should not handle food (90.1%), while 63.9% of participants in a study conducted in Saudi Arabia by Al-Shabib at al. [35] disagree with that statement.

They also show worse behaviour when handling food in situations when they sneeze or cough, while 82.5% of the participants covered their nose and mouth. The results of a study conducted in hospital kitchens in Indonesia [36] are also significant. Hygiene in kitchens where food is prepared for hospitalized patients is important for the health of the patients. The majority of participants (80%) have a good knowledge of health-safe procedures in the process of food handling, 60% of them have acceptable attitudes, while good hygiene practice has been confirmed in 90% of participants. The research by Awad et al. [37] showed a connection between maintaining personal hygiene of employees and microbiological findings of swabs from the hands of employees. All participants washed their hands properly before contact with food, while 93.3% washed their hands properly after food preparation. However, if there are changes on the skin or hands, 20% of them still work with food. In this study, there is no statistically significant difference in knowledge and behaviour among participants in relation to the level of education.

Employees in catering facilities in India were surveyed regarding food hygiene knowledge and behaviour [38], and the majority of employees covered by this survey (82.5%) did not have any type of training, while only 27.9% stated that they heard of foodborne illnesses. A slightly higher level of knowledge was shown by men compared with women, as is the case in some other studies. Most participants acquired knowledge through the media. The results show that most of those involved in the study believe that food contamination takes place through contaminated hands. Most also believe that nail hygiene is very important for preventing food contamination.

### 4.1. Hand Swabs

Hands contamination of food handlers can be used as an indicator of their behaviour regarding food-related practice and personal hygiene. As the participants who participated in this research do not have adequate habits regarding hand hygiene while working with food, the results obtained when sampling hand swabs are not surprising. In restaurants in the examined group, half had the acceptable results of hand swabs, in bakeries 36.6%, and in confectioneries 40.0%. There is no significant difference in the number of unacceptable swabs between the test and control groups. Similar results were obtained in a study conducted in canteens in primary schools in Malaysia [39], where the percentage of unacceptable hand swabs was more than 45%. A study examining the bacterial contamination of the hands of employees who handle food in city restaurants in Sudan [32] obtained very poor results. Namely, 87% of the wipes of the participants’ hands were contaminated. This study showed the need for employees’ education in order to raise their awareness of the importance of personal hygiene, and above all, hand hygiene.

Although the knowledge of the participants after the education increased, their behaviour was not in line with the improvement. After the training, the results were im-proved in the restaurants, where we found 66.6% of acceptable results, and during the third sampling, it was 56.6%; in the control group, the situation was approximately the same. After the training in bakeries, 66.7% were acceptable, which is almost double that of the control group, while in the third sampling, 63.3% were acceptable, and only 36.6% in the control group. In Egypt, similar data were obtained. Namely, of the total number of swabs taken from the hands of kitchen employees, 50% were unacceptable [40]. The most common contaminant of hands in research in Egypt was *Escherichia coli*. It was identified in 41.7% of wipes of employees’ hands before the hand hygiene education; however, after the education, that percentage dropped to 11.1%. Also in this study, the number of *Enterobactericeae* were the most common cause of hand swab malfunctions and unacceptable results, with a prevalence of 58.1%, and after the training, this percentage was not reduced, neither in the examined nor in the control group. Considering that the protocol of taking swabs from the hands of food handlers implies prior hand washing, such results were very unsatisfactory. In addition, if the number of samples and the number of bacterial colonies are taken into account, the results indicate non-compliance with the procedure of proper hand washing, which is a basic hygienic preventive measure when working with food.

Observing the results, there was a positive effect of education, which can be seen through the improvement of swab results during the second sampling; however, that education must be more frequent and continual, as indicated by the results of swabs from the third sampling, after six months. Reasons for the decrease in the percentage of acceptable responses from participants when data were collected after four weeks and then again after six months—aside from insufficient awareness of the importance of food safety and because they do not understand their roles in food safety—can be poor infrastructure, irregular inspection controls, and not organizing a team to supervise food safety in the facility.

### 4.2. Work Surface Swabs

Although the participants showed an exceptional level of knowledge for the maintenance of work surfaces that come into contact with food, the results of work surface swabs showed that they do not apply this in practice. Namely, 66.3% of swabs in restaurants were acceptable in the examined group and 63.3% in the control group. The number of swabs of work surfaces in our study is twice as high as in the research conducted in the area of Lombardy in Italy, where 280 swabs taken from surfaces were analyzed, and a deficiency was found in 17.1% of the samples [41]. After the training, there was an improvement, where 80.0% of the swabs were acceptable, and 73.3% were acceptable six months after the training. There were no significant changes in the control group. The situation is somewhat worse in bakeries, and we find even worse results in pastry shops. Namely, in bakeries, 56.67% in the examined group and 60.00% in the control group were acceptable. After education in the examined group, we have approximately 76% that were acceptable, and in the third sampling, approximately 73% were acceptable, while the results in the control group were worse compared with the first sampling. In pastry shops, slightly more than one-third of the swabs that were taken were acceptable, and the situation was similar in the control group. The number of acceptable swabs increased after training (76.7%), as well as during the third sampling, while in the control group, there are no significant variations. Together, these results indicate that education on food hygiene and safety, as a preventive public health intervention, has had a positive effect.

As with everything that is related to the provision of healthy food, equipment maintenance is an important segment that must be controlled. This is primarily to prevent equipment failure (refrigeration devices, machines for washing dishes, refrigerated cabinets, etc.) during the process of preparation, processing, or serving of food so as not to endanger the health of food. Regular cleaning, washing, and disinfection of equipment are important to avoid cross-contamination of food. Maintenance of equipment is an important prerequisite for maintaining adequate hygiene, for adequate cleaning and disinfection, and for pest control. Poorly maintained equipment, such as refrigeration or heating equipment, can lead to failure to reach the required temperatures necessary for preparing and ensuring healthy food.

The results of a large number of studies examining the impact of the knowledge of employees who work with food on their behaviour are often contradictory. Namely, while the results of a smaller number of studies indicate that better knowledge leads to proper behaviour when working with food, a larger number of studies confirm that a high level of knowledge of hygiene and food safety is not a guarantee for correct behaviour when working with food. Therefore, food handlers should be obliged by food safety regulations to follow the principles of good hygiene practice, regardless of their level of food safety knowledge. The reason for the different results could be related to differences in experience and training of food handlers [42,43]. Good knowledge is important, but putting that knowledge into practice is even more important.

## 5. Conclusions

The behaviour of food handlers, viewed as a whole, is unacceptable. Unacceptable hygienic behaviour was found in relation to hand hygiene. Only half of employees working with food were found to wash their hands properly. The results of this study emphasized the role of food handlers’ behaviour that could constitute a potential risk of foodborne disease outbreaks.

Given that proper hand hygiene is the least expensive and most effective preventive measure in preventing the spread of all infectious diseases, including foodborne diseases, we can be very pleased with the result of training in this regard. This, therefore, underlines the importance of further training to improve food handlers’ knowledge of good hand-washing practices. The recognition of those deficiencies may provide a new approach for the strategic planning and execution of continual training programs from the academic and practical perspectives.

## Figures and Tables

**Table 1:** Socio-demographic data of food handlers participating in the study (*n* = 385).

Variable		*n*	%
Gender	Female	182	47.3
	Male	203	52.7
Age (years)	18–23	36	9.4
	24–28	59	15.3
	29–33	69	17.9
	34–38	65	16.9
	39–43	62	16.1
	44–48	38	9.9
	49–53	24	6.2
	>54	32	8.3
Level of education	Primary	58	15.1
	Secondary	270	70.1
	Tertiary	57	14.8
Work experience (years)	˂1	16	4.2
	1–3	55	14.2
	4–6	55	14.2
	7–9	54	14.0
	10–13	36	9.4
	>13	169	44.0
Work position	Chef	40	10.4
	Cook	95	24.7
	Cook’s assistant	107	27.8
	Manager	20	5.2
	Waiter	63	16.4
	Baker	44	11.6
	Confectioner	16	4.4

**Table 2:** Behaviour of employees before the training (*n* = 385).

Variable	Never		Rarely		Sometimes		Often		Always		% Correct Answers
	*n*	%	*n*	%	*n*	%	*n*	%	*n*	%	
Do you wash your hands for at least 20 s	9	2.3	17	4.4	33	8.6	114	29.6	212	**55.1**	**55.1**
Do you use a towel to wipe your hands after washing	170	**44.2**	50	13.0	66	17.1	44	11.4	55	14.3	**44.2**
Do you use hand sanitizer after washing your hands	33	8.6	19	4.9	83	21.6	76	19.7	174	**45.2**	**45.2**
Do you wash your hands before contact with raw foods	17	4.4	6	1.6	34	8.8	52	13.5	176	**71.7**	**71.7**
Do you wash your hands after contact with raw foods	9	2.3	3	0.8	30	7.8	45	11.7	298	**77.4**	**77.4**
Do you use separate utensils for cooked and raw food	19	4.9	18	4.7	51	13.2	38	9.9	259	**67.3**	**67.3**
Do you cover your mouth and nose when sneezing and coughing	2	0.5	1	0.3	14	3.6	29	7.5	339	**88.1**	**88.1**
Do you wash your hands if you touch your face or hair	15	3.9	19	4.9	43	11.2	52	13.5	256	**66.5**	**66.5**
Do you take off your jewelery (earrings, watch, bracelets, etc.) before you start work	24	6.2	14	3.6	57	14.8	15	13.9	275	**71.4**	**71.4**
Do you ever eat or chew gum while you work	279	**72.5**	22	5.7	61	15.8	0	0.0	23	6.0	72.5
Do you wear headgear	47	12.2	7	1.8	16	4.2	35	9.1	280	**72.7**	**72.7**
Do you smoke while working with food	342	**88.8**	2	0.5	26	6.8	1	0.3	14	3.6	**88.8**

**Table 3:** Behaviour of participants in relation to gender, age, years of service, and education.

Variable	Gender	Age	Years of Service	Education	Catering School	Workplace	Object Type
Do you wash your hands for at least 20 s	0.564	0.391	0.558	0.934	0.550	0.569	0.023 *
Do you use a towel to wipe your hands after washing	0.491	0.388	0.292	0.836	0.880	0.282	0.983
Do you use hand sanitizer after washing your hands	0.492	0.487	0.495	0.834	0.581	0.775	0.181
Do you wash your hands before contact with raw foods	0.869	0.832	0.238	0.928	0.780	0.580	0.004 *
Do you wash your hands after contact with raw foods	0.932	0.816	0.679	0.855	0.856	0.549	0.060
Do you use separate utensils for cooked and raw food	0.809	0.690	0.986	0.918	0.615	0.361	0.044 *
Do you cover your mouth and nose when sneezing and coughing	0.328	0.160	0.030	0.862	0.716	0.472	0.534
Do you wash your hands if you touch your face or hair	0.596	0.815	0.448	0.668	0.032	0.7323	0.083
Do you take off your jewelery (earrings, watch, bracelets, etc.) before you start work	0.164	0.641	0.812	0.705	0.186	0.193	0.327
Do you ever eat or chew gum while you work	0.252	0.823	0.797	0.543	0.527	0.726	0.314
Do you wear headgear	0.499	0.275	0.768	0.203	0.078	0.899	0.368
Do you smoke while working with food	0.856	0.702	0.744	0.512	0.917	0.690	0.943

Legend: the statistically significant differences between the to the observed variables were determined with the χ^2^ square test, * *p* < 0.05—statistically significant difference.

**Table 4:** Behaviour of food handlers before and after the training.

Variables	Before Training	Four Weeks after Training	Six Months after Training
	**Mean ± Sd**	**Mean ± Sd**	**Mean ± Sd**
Do you wash your hands for at least 20 s	55.1 ± 0.96	98.2 ± 0.21	97.6 ± 0.56
Do you use a towel to wipe your hands after washing	44.2 ± 1.48	97.6 ± 0.32	96.2 ± 0.32
Do you use hand sanitizer after washing your hands?	45.2 ± 1.27	92.4 ± 0.56	90.3 ± 0.24
Do you wash your hands before contact with raw foods?	71.7 ± 1.02	99.8 ± 0.12	99.6 ± 0.32
Do you wash your hands after contact with raw foods?	77.4 ± 0.85	98.4 ± 0.23	96.1 ± 0.56
Do you use separate utensils for cooked and raw food?	67.3 ± 1.16	96.4 ± 0.23	90.4 ± 0.47
Do you cover your mouth and nose when sneezing and coughing?	88.1 ± 0.54	99.8 ± 0.12	99.6 ± 0.62
Do you wash your hands if you touch your face or hair?	66.5 ± 1.10	94.8 ± 0.21	93.2 ± 0.32
Do you take off your jewelry (earrings, watch, bracelets, etc.) before you start work?	71.4 ± 1.21	100.0 ± 0.00	86.4 ± 0.61
Do you ever eat or chew gum while you work?	72.5 ± 1.12	99.5 ± 0.21	92.1 ± 0.78
Do you wear headgear?	72.7 ± 1.13	100.0 ± 0.00	98.7 ± 0.24
Do you smoke while working with food?	88.8 ± 0.64	100.0 ± 0.00	96.4 ± 0.10

Legend: Sd—standard deviation.

**Table 5:** TBC of swabs of work surfaces, equipment, and hands of food handlers for restaurants.

Swabs	Before the Training				Four Weeks after the Training				Six Months after the Training			
	Acceptable		Unacceptable		Acceptable		Unacceptable		Acceptable		Unacceptable	
**Examined group**	***n***	**%**	***n***	**%**	***n***	**%**	***n***	**%**	***n***	**%**	***n***	**%**
Working area	19	63.3	11	36.6	24	80.0	6	20.0	22	73.3	8	26.6
Equipment	19	63.3	11	36.6	22	73.3	8	26.6	21	70.0	9	30.0
Hands	15	50.0	15	50.0	20	66.6	10	33.3	17	56.6	13	43.3
**Control group**	***n***	**%**	***n***	**%**	***n***	**%**	***n***	**%**	***n***	**%**	***n***	**%**
Working area	20	66.6	10	33.35	19	63.33	11	36.67	20	66.6	10	33.3
Equipment	19	63.3	11	36.67	20	66.65	10	33.35	20	66.6	10	33.3
Hands	14	46.6	16	53.37	15	50.00	15	50.00	13	43.3	17	56.6

**Table 6:** TBC of swabs of work surfaces, equipment, and hands of food handlers for bakeries.

Swabs	Before the Training				Four Weeks after the Training				Six Months after the Training			
	Acceptable		Unacceptable		Acceptable		Unacceptable		Acceptable		Unacceptable	
**Examined group**	***n***	**%**	***n***	**%**	***n***	**%**	***n***	**%**	***n***	**%**	***n***	**%**
Working area	17	56.6	13	43.3	23	76.6	7	23.3	22	73.3	8	26.6
Equipment	13	43.3	17	56.6	21	70.0	9	30.0	18	60.0	12	40.0
Hands	11	36.6	19	63.3	20	66.7	10	33.3	19	63.3	11	36.6
**Control group**	***n***	**%**	***n***	**%**	***n***	**%**	***n***	**%**	***n***	**%**	***n***	**%**
Working area	18	60.0	12	40.0	18	60.0	12	40.0	13	43.3	17	56.6
Equipment	11	36.6	19	63.3	15	50.0	15	50.0	13	43.3	17	56.6
Hands	9	30.0	21	70.0	10	33.3	20	66.6	11	36.6	19	63.3

**Table 7:** TBC of swabs of work surfaces, equipment, and hands of food handlers for pastry shops.

	Before the Training				Four Weeks after the Training				Six Months after the Training			
	Acceptable		Unacceptable		Acceptable		Unacceptable		Acceptable		Unacceptable	
**Examined group**	***n***	**%**	***n***	**%**	***n***	**%**	***n***	**%**	***n***	**%**	***n***	**%**
Working area	11	36.6	19	63.3	23	76.6	7	23.3	22	73.3	8	26.6
Equipment	18	60.0	12	40.0	22	73.3	8	26.6	20	66.6	10	33.3
Hands	12	40.0	18	60.0	20	66.6	10	33.3	19	63.3	11	36.6
**Control group**	***n***	**%**	***n***	**%**	***n***	**%**	***n***	**%**	***n***	**%**	***n***	**%**
Working area	11	36.6	19	63.3	13	43.3	17	56.6	17	56.6	13	43.3
Equipment	14	46.6	16	53.3	16	53.3	14	46.6	14	46.6	16	53.3
Hands	10	33.3	17	56.6	12	40.0	18	60.0	9	30.0	21	70.0

**Table 8:** Differences in results of swabs analysis in all three types of food facilities.

Food Facility	Before Training	Four Weeks after Training	Six Months after Training	CI	α	*p*
	Mean ± Sd	Mean ± Sd	Mean ± Sd			
Restaurants	41.66 ± 21.0	83.33 ± 8.3	81.66 ± 16.9	95%	0.05	0.000
Bakeries	23.33 ± 24.1	70.00 ± 10.6	60.00 ± 14.6	95%	0.05	0.000
Pastry shop	70.00 ± 11.3	75.00 ± 14.7	70.00 ± 11.3	95%	0.05	0.000

Legend: Sd—standard deviation; CI—confidence interval; α—significance level; *p*—value (association between type of objects and knowledge before and four or six months after training).

**Table 9:** Ratio of swab results for all three samplings.

Dependent/Independent Variable	1/2	1/3	α	CI
Restaurants	0.000	0.000	0.05	95%
Bakeries	0.000	0.000	0.05	95%
Pastry shop	0.000	0.000	0.05	95%

Legend: *p*-value ratio between type of objects; α—significance level; CI—confidence interval.

## Data Availability

The data presented in this study are available on request from the corresponding author.
